# Trametinib sensitizes *KRAS*-mutant lung adenocarcinoma tumors to PD-1/PD-L1 axis blockade via Id1 downregulation

**DOI:** 10.1186/s12943-024-01991-3

**Published:** 2024-04-20

**Authors:** Ander Puyalto, María Rodríguez-Remírez, Inés López, Irati Macaya, Elizabeth Guruceaga, María Olmedo, Anna Vilalta-Lacarra, Connor Welch, Sergio Sandiego, Silvestre Vicent, Karmele Valencia, Alfonso Calvo, Ruben Pio, Luis E. Raez, Christian Rolfo, Daniel Ajona, Ignacio Gil-Bazo

**Affiliations:** 1 Department of Medical Oncology, Cancer Center Clínica Universidad de Navarra (CCUN), Pamplona, Spain; 2 Program in Solid Tumors, Cancer Division, Cima Universidad de Navarra, CCUN, Av. Pio XII, 55, 31008 Pamplona, Spain; 3 Instituto de Investigación Sanitaria de Navarra (IDISNA), Pamplona, Spain; 4 Centro de Investigación Biomédica en Red de Cáncer (CIBERONC), Madrid, Spain; 5 Bioinformatics Platform, Cima Universidad de Navarra, Pamplona, Spain; 6 Department of Oncology, Fundación Instituto Valenciano de Oncología (FIVO), C/Beltrán Báguena 8. 46009, Valencia, Spain; 7 Department of Biochemistry and Genetics, School of Sciences, Universidad de Navarra, Pamplona, Spain; 8 Memorial Cancer Institute, Memorial Healthcare System, Florida Atlantic University (FAU), Pembroke Pines, FL USA; 9 Center for Thoracic Oncology, Tisch Cancer Institute, Mount Sinai Health System, New York, USA

**Keywords:** *KRAS*-mutant lung adenocarcinoma, Trametinib, Id1, PD-1 inhibition, PD-L1, Proteasome

## Abstract

**Background:**

The identification of novel therapeutic strategies to overcome resistance to the MEK inhibitor trametinib in mutant *KRAS* lung adenocarcinoma (LUAD) is a challenge. This study analyzes the effects of trametinib on Id1 protein, a key factor involved in the *KRAS* oncogenic pathway, and investigates the role of Id1 in the acquired resistance to trametinib as well as the synergistic anticancer effect of trametinib combined with immunotherapy in *KRAS*-mutant LUAD.

**Methods:**

We evaluated the effects of trametinib on *KRAS*-mutant LUAD by Western blot, RNA-seq and different syngeneic mouse models. Genetic modulation of Id1 expression was performed in *KRAS*-mutant LUAD cells by lentiviral or retroviral transductions of specific vectors. Cell viability was assessed by cell proliferation and colony formation assays. PD-L1 expression and apoptosis were measured by flow cytometry. The anti-tumor efficacy of the combined treatment with trametinib and PD-1 blockade was investigated in *KRAS*-mutant LUAD mouse models, and the effects on the tumor immune infiltrate were analyzed by flow cytometry and immunohistochemistry.

**Results:**

We found that trametinib activates the proteasome-ubiquitin system to downregulate Id1 in *KRAS*-mutant LUAD tumors. Moreover, we found that Id1 plays a major role in the acquired resistance to trametinib treatment in *KRAS*-mutant LUAD cells. Using two preclinical syngeneic *KRAS*-mutant LUAD mouse models, we found that trametinib synergizes with PD-1/PD-L1 blockade to hamper lung cancer progression and increase survival. This anti-tumor activity depended on trametinib-mediated Id1 reduction and was associated with a less immunosuppressive tumor microenvironment and increased PD-L1 expression on tumor cells.

**Conclusions:**

Our data demonstrate that Id1 expression is involved in the resistance to trametinib and in the synergistic effect of trametinib with anti-PD-1 therapy in *KRAS*-mutant LUAD tumors. These findings suggest a potential therapeutic approach for immunotherapy-refractory *KRAS*-mutant lung cancers.

**Graphical Abstract:**

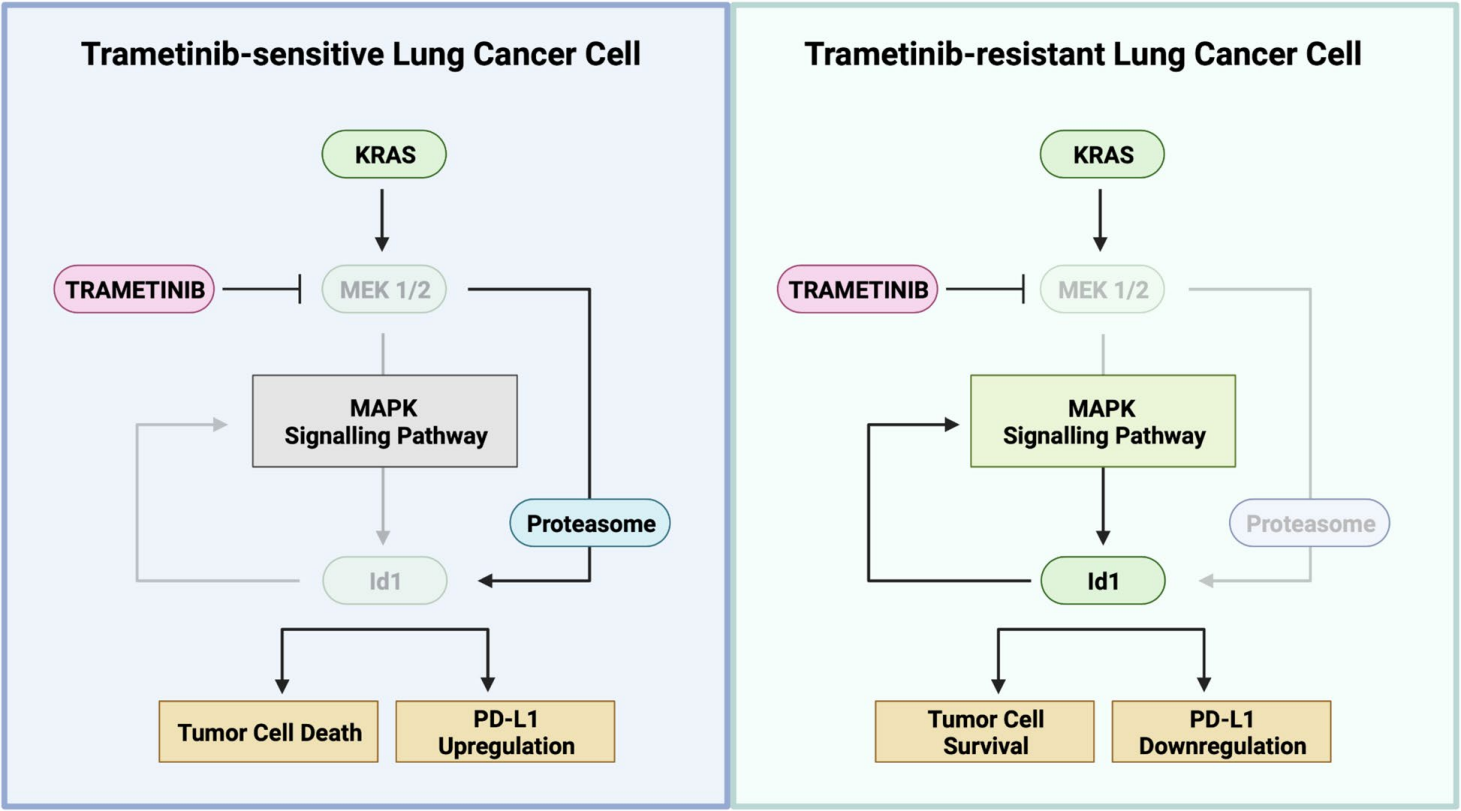

**Supplementary Information:**

The online version contains supplementary material available at 10.1186/s12943-024-01991-3.

## Background

Lung cancer is the leading cause of cancer death worldwide [1]. Genetic characterization of oncogenic driver mutations (*EGFR*, *KRAS*, *ALK*, *ROS*) has transformed the management of non-small cell lung cancer (NSCLC) therapy with the introduction of molecularly targeted therapies, allowing an individualized treatment approach [2, 3]. *KRAS* mutations represent the most prevalent genetic driver alteration in lung adenocarcinomas (LUAD) [2]. Despite the approval of KRAS^G12C^ inhibitors, adagrasib (MRTX849) and sotorasib (AMG510) for NSCLC patients harboring *KRAS*^*G12C*^ mutations, *KRAS* alterations are still related to unfavorable therapeutic outcomes and improvement in survival rates in this LUAD subtype remains an unmet clinical need [4, 5].

Targeting *KRAS* signaling at the level of its downstream effectors, such as mitogen-activated protein kinases (MAPK), has also been studied [6]. Trametinib (GSK1120212) is an oral, reversible and selective allosteric inhibitor of MEK1/2 and is FDA-approved for the treatment of metastatic *BRAF*^*V600E*^ mutant NSCLC and melanoma, in combination with BRAF inhibitors [7, 8]. However, the antitumor activity of MEK1/2 inhibitors in monotherapy or in combination with conventional chemotherapy has been demonstrated to be ineffective in a significant proportion of patients with *KRAS*-mutant advanced NSCLC [9, 10]. Therefore, diverse combination therapies employing targeted therapies or monoclonal antibodies, such as programed-cell death protein 1 (PD-1)/ programed-cell death-ligand 1 (PD-L1) inhibitors, are emerging in an attempt to overcome primary drug resistance and/or acquired resistance to trametinib [11, 12].

Immune checkpoint inhibitors (ICIs) have shown unprecedented clinical results in advanced NSCLC, providing durable responses and prolonging long-term survival [13]. However, only a small subset of *KRAS*-mutant lung cancer patients obtains a significant long-term benefit from these treatments [14–16]. These drugs reactivate antigen-specific antitumor effector T cells, boosting the immune response against cancer cells. A range of studies have demonstrated that molecularly targeted therapies can modulate tumor antigenicity and potentiate T cell immune recognition, improving the efficacy of ICIs [17, 18]. Recently, preclinical studies in *KRAS*-mutant murine LUAD models demonstrated the therapeutic benefit of combining *inhibitor of differentiation-1* (*Id1)* blockade with ICIs. *Id1* gene silencing enhanced PD-L1 expression on lung cancer cells, sensitizing tumor cells to PD-1/PD-L1 axis blockade and enhancing CD8^+^ T cell infiltration [19]. *Id1* is a member of the inhibitor of differentiation (*Id1*-*Id4*) family. Id proteins, which are described as dominant negative transcriptional regulators, are overexpressed in human cancers, including lung malignancies [20, 21]. In the *KRAS*-mutant LUAD setting, *Id1* expression, known to provide an aggressive pro-oncogenic phenotype and to enhance the colonization capacity of lung cancer cells, has been shown to correlate with reduced overall survival and poor treatment response [22–24].

Based on these premises, we aimed to evaluate the effect of trametinib in *Id1* in different *KRAS*-mutant LUAD cells and preclinical in vivo models. Here we provide key evidence on the preclinical and functional role of *Id1*, sustaining MAPK (KRAS-MEK1/2) signaling in the acquired resistance to trametinib and PD-1/PD-L1 blockade in *KRAS*-mutant LUAD tumors via PD-L1 downregulation.

## Methods

### Cell lines and reagents

Human LUAD cell lines (H1792, H2009, H23, A549, H2030, H1437, H1568, and H2126) and murine LUAD cell line Lewis Lung Carcinoma (LLC) were purchased from the American Type Culture Collection. KLA LUAD cells were derived from *KR181*^fl/fl^ mice [25]. 393P cells were derived from *KRAS*^*LA1/*+^*; p53*^*R172HDG*^ mice, and provided by Dr. J. M. Kurie (The University of Texas, MD Anderson Cancer Center, Houston, TX, USA). Lacun3 LUAD cell line, which was established from a chemically induced LUAD mice model [26], was a gift of Prof. L. Montuenga (Cima Universidad de Navarra, Pamplona, Spain). CMT167 LUAD cell line was kindly provided by Dr. F. Torres Andon (Center for Research in Molecular Medicine and Chronic Diseases, Santiago de Compostela, Spain). Murine and human cells were cultured at 37 °C and 5% CO_2_ in DMEM (Gibco, Waltham, MA, USA) or RPMI 1640 medium (Gibco, Waltham, MA, USA) supplemented with 10% fetal bovine serum and 1% Penicillin/Streptomycin (Gibco, Waltham, MA, USA). Cells were routinely tested for *Mycoplasma* with the MycoAlert Mycoplasma Detection Kit (Lonza, Basel, Switzerland).

Trametinib, GSK2126458 and proteasome inhibitor (MG-132) #S2619 were acquired from Selleck chemicals (Houston, TX, USA). Trametinib was resuspended in dimethyl sulfoxide (DMSO) (Sigma-Aldrich, MO, USA) for in vitro experiments and in corn oil (Sigma-Aldrich, MO, USA) for in vivo assays. Hydroxychloroquine sulfate H0915 (CQ) MFCD00078203 was obtained from Sigma-Aldrich (MO, USA).

Trametinib-resistant (TR) cell lines were generated by culturing murine (CMT167, LLC and KLA) and human (H1792, H2009, A549 and H2030) parental lines with increasing concentrations of trametinib up to 500 nM, as previously described [27]. TR cell lines were always cultured in cell media with trametinib (500 nM) which was renewed every 4 days. TR-cell resistance to trametinib was measured by analyzing the proliferation capacity of these cells in the presence of trametinib, and compared with parental cells.

### In vitro assays

*Id1* silencing was performed as previously described [19, 23, 24]. The expression of Id1 was upregulated by lentiviral transduction with mouse or human Id1-flag cDNA expressing vectors (Id1-flag) or retroviral transduction of human Id1 cDNA (Id1-OE) as previously described [19, 24]. Transduction with a GFP cDNA expressing vector was used as control. Oligonucleotides for SMURF2 shRNA (TRCN0000027749) were: Forward sequence: 5'-CCGGCCACACTTGCTTCAATCGAATCTCGAGATTCGATTGAAGCAAGTGTGGTTTTTG-3' Reverse sequence: 5'-AATTCAAAAACCACACTTGCTTCAATCGAATCTCGAGATTCGATTGAAGCAAGTGTGG-3' (Sigma-Aldrich, MO, USA). Oligonucleotides were annealed and cloned into the lentiviral plasmid pLKO (Addgene plasmid #21,915) (MA, USA).

Western blot analysis were performed as previously described [23]. The antibodies used and their dilutions are indicated in Supplementary Table S1. The quantification of the Western blot band intensity was done using Image Lab software (Bio-Rad, CA, USA), and normalized with the density of the loading control protein. RNA extraction and real-time PCR were performed as previously described [23]. The sequences of the primers used in the real-time PCR assays are indicated in Supplementary Table S2.

For proliferation assays, 2500 cells per well were seeded in 96-well plates in 100 μL of complete medium in triplicate for each experimental condition. Cell proliferation was assessed at 72 h using trametinib concentrations ranging from 2000 nM to 0.008 nM. Cells were fixed with formaldehyde and stained with a commercial 1% crystal violet solution (V5265, Sigma-Aldrich, Saint Louis, MO, USA). A 20% acetic solution was used to dissolve crystal violet, and the absorbance was measured at 590 nm in a SPECTROstar Nano (BGM LABTECH, Ortenberg, Germany).

### RNA sequencing

RNA sequencing data analysis was performed using the following workflow: (1) the quality of the raw reads was verified using FastQC software and the trimming of the reads was carried out with trimmomatic [28]; (2) alignment against the mouse reference genome (GRCh38) was performed using STAR [29]; (3) gene expression quantification using read counts of exonic gene regions was carried out with featureCounts [30]; (4) the gene annotation reference was Gencode v42 [31]; and (5) differential expression statistical analysis was performed using R/Bioconductor (https://www.R-project.org/) as follows. Gene expression data was normalized with edgeR [32] and voom [33]. After quality assessment and outlier detection using R/Bioconductor, a filtering process was performed. Genes with read counts lower than 4 in more than the 50% of the samples of all the studied conditions were considered as not expressed. LIMMA was used to identify the genes with significant differential expression between experimental groups. Genes were selected as differentially expressed using a cut-off value of B > 5. Further functional and clustering analyses and graphical representations were performed using the R/Bioconductor and clusterProfiler [34]. Data are publicly available in GEO database with the accession number GSE236258.

### Mouse lung cancer models and therapeutic schedules

All animal procedures were approved by the institutional Committee on Animal Research and Ethics (regional Government of Navarra) under the protocol numbers E17-22(054-19E2), E18-21(054-19E2) and E24-20(049-E1). This study included 8–12 week-old female C57BL/6 J mice (Envigo, Indianapolis, IN, USA) and 8 week-old Sv/129 female mice (Janvier Labs, Le Genest-Saint-Isle, France).

LLC cells (1.5 × 10^6^), LLC cells expressing Id1-flag (1.5 × 10^6^) or CMT167 cells (2 × 10^5^) were subcutaneously inoculated in the right flank of syngeneic mice. LLC and CMT167 engraftments were allowed to grow for 7 days, and then treated with vehicle or with trametinib (1 mg/kg; oral administration, 5 times a week) until day 30.

To assess the antitumor effect of trametinib and its combination anti-PD-1, LLC or 393P (4 × 10^6^) cells were subcutaneously injected in the right flank of female C57BL/6 J and Sv/129 mice, respectively. LLC and 393P tumors were allowed to grow for 7 and 14 days before treatment, respectively. Tumor-bearing mice were randomized in four groups and treated with anti-PD-1 (100 mg/mouse; intraperitoneally, twice a week; RMP1-14 BioXCell, Lebanon, NH, USA), trametinib (1 mg/kg; oral oral administration, 5 times a week), their combination, or vehicle for three weeks.

Tumors were measured periodically using a digital caliper (DIN862, Ref 112-G, SESA Tools, Hernani, Spain), and tumor volume was calculated using the following formula:

$$\mathrm{Tumor}\;\mathrm{Volume}\:=\:\mathrm\pi/6\:\;\times\;\:\mathrm{length}\;\;\times\;\mathrm{width}^2$$

### Immunohistochemistry (IHC)

For histological analyses, tumors were harvested, fixed in formaldehyde 4% pH = 7 (Panreac, Castellar del Valles, Spain) for 48 h, embedded in paraffin, and sectioned for hematoxylin–eosin staining (H&E). Immunohistochemistry (IHC) for the detection of Id1, CD8, CD3, CD4 and FOXP3 was performed as previously described [19, 24]. Slides were scanned with the Aperio Digital Scanner (Leyca, Wetzlar, Germany) and analyzed with ImageJ (NIH, Bethesda, MD, USA).

### Flow cytometry analyses

Tumors were dissected, cut into small pieces, and incubated with 1 mg/ml collagenase D and 50 µg/ml DNase I (Roche, Basel, Switzerland) at 37 °C for 30 min. EDTA (6 µM) was then used to block collagenase and DNase I activities. Then, tumors were mechanically disaggregated through a 70 μm cell strainer (Corning, NY, USA). Erythrocytes were lysed with ACK buffer (Gibco, Waltham, MA, USA). Single cell suspensions were layered over 35% Percoll (GE Healthcare, IL, USA) and centrifuged to purify the tumor infiltrating leukocytes for flow cytometry analysis. Cell suspensions were incubated with a purified monoclonal antibody against CD16/CD32 (Mouse BD Fc Block, 1:200, BD Bioscience, NJ, USA) for 15 min at 4ºC and stained with fluorochrome-conjugated antibodies diluted in FACS buffer (PBS, 5% Fetalclone, 2.5 mM EDTA). For intracellular staining, cells were permeabilized with eBioscience Fixation/Permeabilization Kit (Invitrogen, Waltham, MA, USA) for 15 min and stained. The antibodies used for flow cytometry analyses are listed in Supplementary Table S3. Dead cells were excluded using PromoFluor-840 NIR Maleimide (PromoCell, Heidelberg, Germany). Gating strategies are shown in Supplementary Figs. S1 and S2. 

Quantification of apoptosis was assessed by flow cytometry as previously described [19]. PD-L1 expression was assessed by flow cytometry, as previously described [2]. Briefly, LUAD cells were seeded into 6-well plates. After 24 h, human and murine lung cancer cells were treated with trametinib for 72 h. Trametinib concentrations used were 100 nM for murine and 500 nM for human LUAD cells. Control cells were treated with DMSO. After 48 h of incubation, recombinant murine (500 U/mL, PeproTech, MA, USA) or human (20 ng/ml, Biolegend,CA, USA) IFN-γ was added, and the cells were incubated for 24 h. Cells without IFN-γ were also analyzed. PE-conjugated anti-CD274 (PD-L1) was used to detect PD-L1 expression on lung cancer cell surface. Cells were acquired using a CytoFLEX LX flow cytometer (Beckman Coulter, CA, USA) and analyzed with CytExpert software (Beckman Coulter, CA, USA).

### Statistical analysis

Data normality was assessed using the Shapiro–Wilk test. Parametric comparations between experimental groups were performed by two-sided t-test or one-way ANOVA test followed by the Tukey post hoc test. Non-parametric comparisons between experimental groups were performed by two-sided Mann–Whitney *U*-test or Kruskal–Wallis test with post hoc Mann–Whitney *U*-test. Survival curves were generated using the Kaplan–Meier method, and differences were analyzed with the log-rank test. *p* < 0.05 was considered statistically significant. Statistical analyses were performed using the version 9 of GraphPad Prism software (GraphPad Prism 9, San Diego, CA, USA).

## Results

### Trametinib reduces Id1 levels in NSCLC cells in vitro and in vivo

**Fig. 1:**
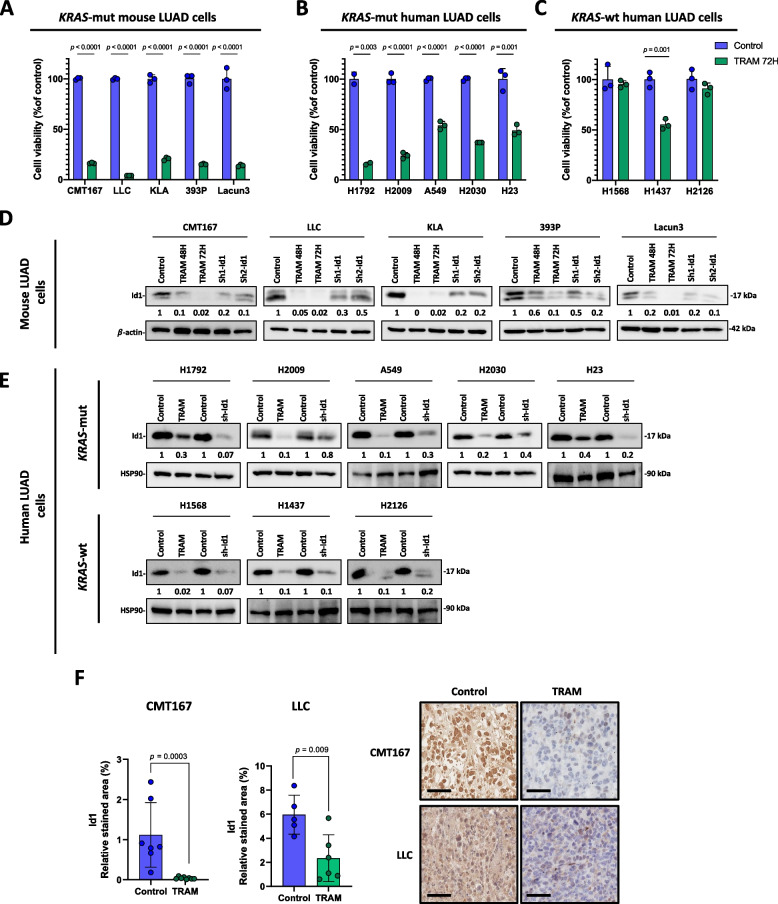
Trametinib reduces Id1 protein levels in *KRAS*-mutant LUAD in vitro and in vivo. **A** Cell viability assay of *KRAS*-mutant mouse LUAD cells treated with trametinib (TRAM) 100 nM for 72 h. **B** Cell viability of *KRAS*-mutant human LUAD cells treated with trametinib (TRAM) 500 nM for 72 h. **C** Cell viability of *KRAS*-wild type human LUAD cells treated with trametinib (TRAM) 500 nM for 72 h. **D** Western blot analysis of Id1 protein in *KRAS*-mutant mouse LUAD cells treated as in A. ß-actin was used as control. **E** Western blot analysis of Id1 in human *KRAS*-mutant (upper panel) and in human *KRAS*-wild type LUAD cells (lower panel) treated as in B-C. HSP90 was used as control. **F** Immunohistochemical quantification of Id1 expression in CMT167 and LLC tumors harvested at day 19 from mice treated with vehicle (Control) or trametinib (TRAM). Left panel: Barr graphs showing the Id1 expression. Right panel: Representative Id1 immunohistochemical stainings. In Western blot analyses, relative optical density is indicated underneath each lane. Data are expressed as mean ± SD. Comparisons between experimental groups were performed by two-sided *t*-test (parametric) or two-sided Mann–Whitney *U*-test (non-parametric)

The constitutive activation of the MAPK pathway as a result of *KRAS* mutations has led to the use of trametinib as a potential therapeutic option to treat *KRAS*-mutant cancers [35]. We first assessed the cytotoxic effect of trametinib in a panel of murine and human lung cancer cells. A significant impairment of cell growth was observed in all murine and human *KRAS*-mutant LUAD cell lines (Fig. 1A and B). Nonetheless, proliferation of *KRAS*-wild type lung cancer cells seemed not to be affected by trametinib (Fig. 1C). Among the *KRAS*-wild type lung cancer cell lines analyzed, trametinib only impaired the viability of the H1437, a cell line that harbors *MAP2K1* mutation [36]*.* These observations suggest that the antitumor activity of trametinib is mainly associated with *KRAS* mutational status.

Previously, we found that *Id1* is a key factor of the MAPK signaling pathway in *KRAS*-mutant human LUAD [24]. Interestingly, trametinib treatment diminished Id1 protein levels in *KRAS*-mutant and *KRAS*-wildtype LUAD cells. The magnitude of this downregulation was even higher than the observed with a short hairpin (sh) RNA targeting *Id1* (sh-*Id1*) in most of cell lines studied (Fig. 1D and E).

In view that trametinib treatment downregulates Id1 expression, we analyzed the effects of this treatment on Id1 expression and tumor growth in CMT167 and LLC *KRAS*-mutant LUAD syngeneic tumors. Trametinib treatment significantly reduced CMT167 and LLC tumor volumes [Tumor Volume (mm^3^): CMT167: Control: 12.86 ± 3.570, CMT167 TRAM: 6.976 ± 1.771; LLC: Control: 35.45 ± 7.648, TRAM: 16.08 ± 3.88) (Supplementary Fig. S3). Consistently with the effects of trametinib on Id1 expression in vitro, IHC analysis revealed a downregulation of Id1 expression in CMT-167 and LLC tumor tissues in vivo (CMT167: Control: 1.12 ± 0.3038, CMT-167 TRAM: 0.04059 ± 0.0097; LLC: Control: 5.96 ± 1.617, TRAM: 2,339 ± 1.946) (Fig. 1F).

Overall, these data suggest that trametinib reduces Id1 levels in vitro and in vivo in *KRAS*-mutant LUAD, and impairs lung cancer tumor growth.

### Trametinib enhances proteasome activity to degrade Id1

**Fig. 2:**
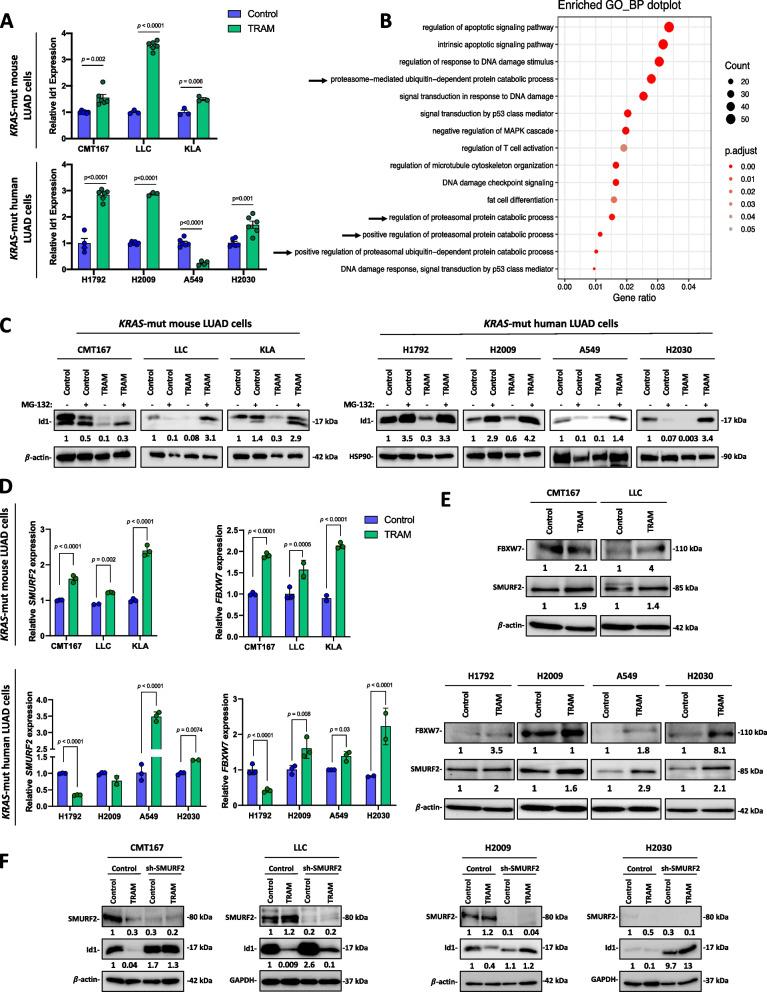
Trametinib increases proteasome activity to reduce *Id1* levels in *KRAS*-mutant LUAD cells. **A** *Id1* mRNA expression levels assessed by real-time PCR in *KRAS*-mutant mouse (upper panel) and human (lower panel) LUAD cells treated with trametinib (TRAM) (100 nM in murine cells and 500 nM in human cells) for 72 h or vehicle (Control). *β-ACTIN* was used as the reference gene. **B** GO Biological process (BP) enrichment analysis of murine *KRAS*-mutant LUAD cells (CMT167 and KLA) treated with trametinib (TRAM) (100 nM) for 72 h or vehicle (Control). **C** Western blot analysis of Id1 protein levels in *KRAS*-mutant mouse (left panel) and human (right panel) LUAD cell lines treated with trametinib (TRAM) (100 nM in murine cells and 500 nM in human cells) for 72 h or vehicle (Control), in the presence or not of the proteasome inhibitor MG-132 (5 μM; 6 h). ß-actin and HSP90 were used as controls for mouse and human cell lines, respectively. **D** E3 ubiquitin ligases *SMURF2* and *FBXW7* mRNA expression levels assessed by real-time PCR in *KRAS*-mutant mouse (upper panel) and human (lower panel) LUAD cells treated with (TRAM) (100 nM in murine cells and 500 nM in human cells) for 6 h or vehicle (Control). *β-ACTIN* was used as the reference gene. **E** Western blot analysis of the E3 ubiquitin ligases SMURF2 and FBXW7 in *KRAS*-mutant mouse (upper panel) and human (lower panel) LUAD cell lines treated with trametinib (TRAM) (100 nM in murine cells and 500 nM in human cells) for 6 h or vehicle (Control). β-actin was used as control. **F** Western blot analysis of the E3 ubiquitin ligase SMURF2 and Id1 in *KRAS*-mutant mouse (left panel) and human (right panel) LUAD cell transduced with lentiviral shRNAs targeting *SMURF2* or with a scrambled sequence (Control). Cells were treated with trametinib (TRAM) (100 nM in murine cells and 500 nM in human cells) for 72 h or vehicle (Control). GAPDH and β-actin were used as control. In Western blot analyses, relative optical density is indicated underneath each lane. Data are expressed as mean ± SD. Comparisons between experimental groups were performed by two-sided *t*-test (parametric) or Mann–Whitney *U*-test (non-parametric)

In order to investigate the molecular mechanisms involved in the trametinib-mediated Id1 protein downregulation, we first analyzed by real-time PCR the effects of trametinib on *Id1* mRNA relative expression in a panel of mouse and human trametinib-treated *KRAS*-mutant LUAD cells. In contrast to the effects observed for Id1 protein levels, an increase in *Id1* mRNA levels were observed in most *KRAS*-mutant trametinib-treated cells (Fig. 2A). This suggests that trametinib treatment is associated with a post-translational downregulation of Id1 protein.

A transcriptomic profiling using RNA sequencing (RNA-seq) data was performed on murine CMT167 and KLA lung cancer cells treated or not with trametinib. Functional analyses (GO) identified a significant enrichment of pathways related with the proteasome-ubiquitin system in trametinib-treated cells compared with control cells (Fig. 2B). The proteasome system regulates intracellular protein degradation, so it may be involved in trametinib-mediated Id1 inhibition [37]. Remarkably, proteasome blockade using a specific inhibitor (MG-132) abolished trametinib-mediated Id1 downregulation in mouse as well as in human *KRAS*-mutant LUAD cells (Fig. 2C). Of note, CQ, an autophagy inhibitor, did not affect trametinib-mediated Id1 inhibition in trametinib-treated mouse and human *KRAS*-mutant LUAD cells [38], suggesting that autophagy was not involved in trametinib-mediated Id1 downregulation (Supplementary Fig. S4A).

Previous studies demonstrate that E3 ubiquitin ligases mediate ubiquitination and degradation of Id family members [39, 21]. RNA-seq analysis on murine CMT167 and KLA *KRAS*-mutant LUAD cells treated or not with trametinib demonstrated that trametinib upregulates the expression of SMURF-2 and FBXW7 E3 ubiquitin ligases (Supplementary Fig. S4B). These data were validated by real-time PCR and Western blot analysis in human as well as in mouse *KRAS*-mutant LUAD cells (Fig. 2D and E). Interestingly, sh-mediated silencing of SMURF2 restored Id1 protein levels in trametinib-treated human as well as murine *KRAS*-mutant LUAD cells (Fig. 2F). This result suggests that trametinib-mediated Id1 downregulation is induced, at least in part, by SMURF2-mediated proteasome-dependent degradation.

### Id1 is associated with the acquired resistance to trametinib in *KRAS*-mutant NSCLC cells

The failure of trametinib in clinical trials performed in patients with *KRAS*-mutant NSCLC has been determined mainly by the appearance of acquired resistance [9]. The identification of the biological mechanisms implicated in resistance to MEK inhibition has been stablished as a strategy to overcome the resistance to these inhibitors [40]. To analyze such mechanisms, murine (CMT167, LLC and KLA) and human (H1792, H2009, A549 and H2030) trametinib resistant (TR) *KRAS*-mutant LUAD cells were generated as previously described [27] and tumor cells were considered TR when their trametinib IC50 at 72 h became significantly higher than the IC50 of parental cells treated with trametinib [41] (Supplementary Fig. S5A).

**Fig. 3:**
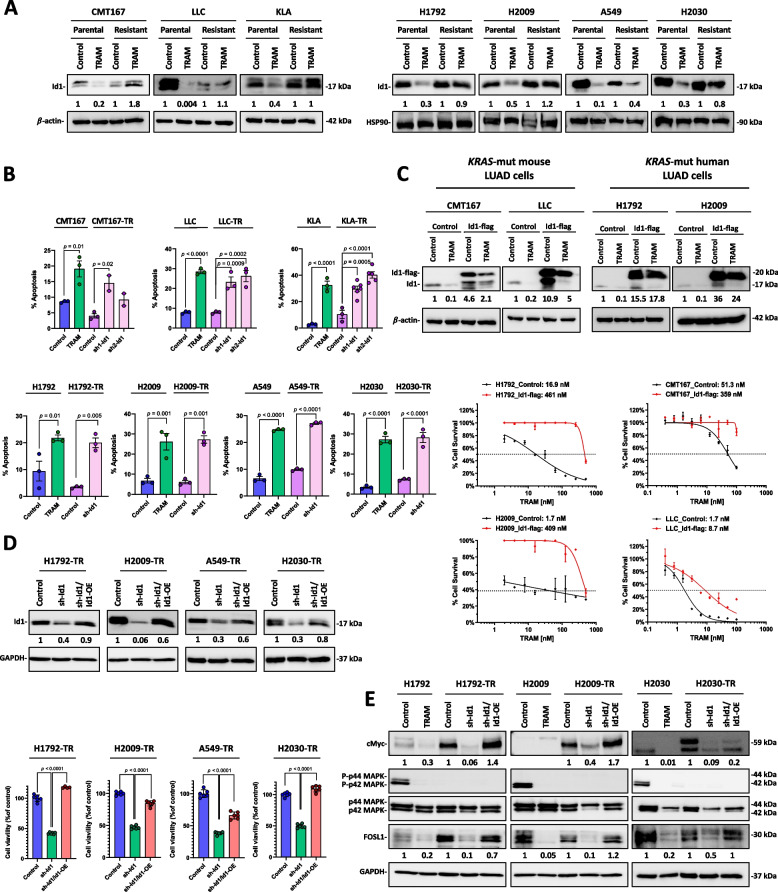
Id1 is involved in the acquired resistance of *KRAS*-mutant LUAD cells to trametinib treatment. **A** Western blot analysis of Id1 protein in parental and trametinib resistant (TR) *KRAS*-mutant mouse (left panel) and human (right panel) cells lines. Cells treated with trametinib (TRAM) (100 nM in murine cells and 500 nM in human cells) for 72 h or vehicle (Control). ß-actin and HSP90 were used as controls for mouse and human cell lines, respectively. **B** Flow cytometry analysis of the proportion of apoptotic parental and TR *KRAS*-mutant mouse (upper panel) and human (lower panel) cells. Parental cells were treated with trametinib (TRAM) (100 nM in murine cells and 500 nM in human cells) for 72 h or vehicle (Control). Id1 was silenced in TR cells using lentiviral transduced shRNAs (sh1-Id1 and sh2-Id1 in mice and sh-Id1 in human) or with a scrambled sequence (Control) TR-cell lines were cultured in cell media with trametinib (500 nM). Apoptosis was assessed by flow cytometry by Annexin V and 7AAD staining, as previously described [19, 24]. **C** Upper panel: Western blot analysis of Id1 (17 kDa) and Id1-flag (20 kDa) proteins. Control cells were transfected with a GFP cDNA expressing vector (control). Cells were treated with trametinib (TRAM) (100 nM in murine cells and 500 nM in human cells) for 72 h or vehicle (Control). β-actin was used as control. Lower panel: Effects of exogenous Id1 (Id1-flag)-transduction in the survival of *KRAS*-mutant mouse (CMT167, LLC) and *KRAS*-mutant human (H1792 and H2009) trametinib-treated tumor cells. Trametinib IC50 is indicated in the figure. **D** Upper panel: Western blot analysis of Id1 protein in TR *KRAS*-mutant human cells lines. GAPDH was used as control. Id1 was silenced using the sh1-Id1. The expression of Id1 in sh1-Id1-transduced cells was rescued by retroviral transduction of Id1 cDNA refractory to sh-Id1 inhibition (sh1-Id1/Id1-OE) [24]. Cells treated with Trametinib (TRAM) 500 nM for 72 h or vehicle (Control). Lower panel: Cell viability of the upper panel cells when treated with trametinib (TRAM) 500 nM for 72 h or vehicle (Control). **E** Western blot analysis of c-Myc, total and phosphorylated p42 MAPK, total and phosphorylated p44 MAPK and FOSL1 in parental and in TR *KRAS*-mutant human cells lines treated as shown in the upper panel of D. GAPDH was used as control. In Western blot analyses, relative optical density is indicated underneath each lane. Data are expressed as mean ± SD. Comparisons between experimental groups were performed by one-way ANOVA followed by the Tukey post hoc test

Reactivation of the MAPK pathway has been reported as a mechanism of acquired resistance to MEK1/2 inhibition [40]. In view that Id1 is a key factor involved in this pathway [24], we studied the effects of trametinib on the Id1 protein expression levels in murine and human TR *KRAS*-mutant LUAD cells. In contrast to the effect observed in parental cell lines, Id1 protein levels were not reduced in TR cells (Fig. 3A). Interestingly, the RNA-seq analysis of parental CMT167 cells and TR CMT167 cells indicated a downregulation of *SMURF2* and upregulation of *Id1* mRNA in TR cells when compared with their parental-treated counterparts (Supplementary Fig. S5B). Next, we explored the potential contribution of Id1 to the acquired resistance to trametinib-mediated apoptosis, a core mechanism of trametinib-mediated cancer cell death [42, 43], in TR *KRAS*-mutant mouse and human LUAD cells. In the presence of trametinib, the proportion of apoptotic cells was significantly lower in TR cells when compared to their parental counterparts in all the *KRAS*-mutant mouse and human LUAD cell lines analyzed. Importantly, the inhibition of *Id1* by specific shRNAs (sh1-Id1 and sh2-Id1 in mice and sh-Id1 in human) (Supplementary Fig. S5C) in TR cells restored the levels of trametinib-mediated apoptosis (Fig. 3B).

To evaluate the functional implication of Id1 in trametinib acquired resistance, exogenous Id1 (Id1-flag) was expressed in *KRAS*-mutant mouse (CMT167, LLC and 393P) and human (H1792 and H2009) LUAD cells. Overall, the magnitude of the trametinib-mediated Id1 reduction was markedly lower in cell lines with Id1-flag expression when compared with their GFP-expressing counterparts (Control) (Fig. 3C; Supplementary Fig. S5D). Importantly, Id1-flag expression rendered *KRAS*-mutant mouse and human LUAD cells resistant to trametinib treatment (Fig. 3C; Supplementary Fig. S5D), suggesting a role of Id1 in the acquired resistance to trametinib treatment. Consistently, the inhibition of *Id1* expression by a lentiviral shRNA (sh-Id1) significantly reduced the viability of TR *KRAS*-mutant human LUAD cells. This viability was rescued when the levels of *Id1* were restored by retroviral transduction of *Id1* cDNA refractory to sh-Id1 (Id1-OE) in TR *KRAS*-mutant human LUAD sh-Id1 cells (Fig. 3D). Moreover, the rescue of *Id1* levels in human TR *KRAS*-mutant LUAD sh-Id1 cells also enhanced the protein levels of FOSL1 and cMyc in the absence of ERK1/2 activation (Fig. 3E), which are associated with the *KRAS* oncogenic pathway and/or have been associated with trametinib resistance [24, 44, 45].

Overall, these data suggest a prominent role of Id1 in the acquired resistance of LUAD cells to trametinib treatment.

### Trametinib enhances IFN-γ-mediated PD-L1 expression and sensitizes *KRAS*-mutant LUAD tumors to anti-PD-1 blockade

**Fig. 4:**
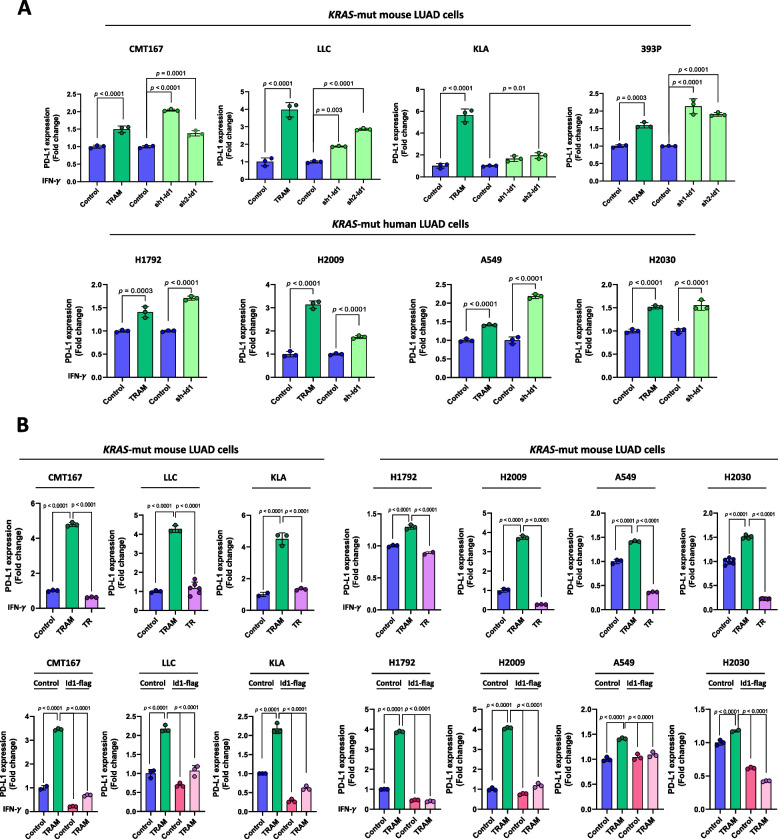
Trametinib upregulates IFN-γ-mediated PD-L1 expression through Id1 downregulation in *KRAS*-mutant LUAD cells. **A** PD-L1 expression assessed by flow cytometry in *KRAS*-mutant mouse (upper panel) and human (lower panel) cells lines transduced or not with lentiviral shRNAs targeting Id1 (sh1-Id1 and sh2-Id1 in mice and sh-Id1 in human). **B** Upper panel: PD-L1 expression assessed by flow cytometry in parental and TR *KRAS*-mutant mouse (left) and human (right) cells lines. Parental cells were treated with trametinib (TRAM) (100 nM in murine cells and 500 nM in human cells) for 72 h or vehicle (Control). TR-cell lines were cultured in cell media with trametinib (500 nM). Lower panel: PD-L1 expression assessed by flow cytometry in *KRAS*-mutant mouse (left) and human cells lines (right) transduced with an Id1-flag cDNA expressing vector (Id1-flag) or a GFP cDNA expressing vector (Control). Cells were treated with trametinib (TRAM) (100 nM in murine cells and 500 nM in human cells) for 72 h or vehicle (Control). Mouse cells were incubated or not with murine IFN-γ (1500 U/ml) for 24 h and human cells were incubated or not with human IFN-γ (20 ng/ml) for 24 h. Data are expressed as mean ± SD. Comparisons between experimental groups were performed by one-way ANOVA test followed by the Tukey post hoc test

Previously, we found that *Id1* silencing was significantly associated with an increase of IFN-γ-mediated PD-L1 expression in both murine and human *KRAS*-mutant LUAD cells [19]. Trametinib treatment enhanced the expression of PD-L1 upon IFN-γ stimulation in *KRAS*-mutant LUAD cells (Fig. 4A). Similar results were obtained when Id1 expression was inhibited by lentiviral transduction with shRNAs targeting Id1 (sh1-Id1 and sh2-Id1 in mice and sh-Id1 in human) (Fig. 4A). Interestingly, in TR *KRAS*-mutant LUAD cells IFN-γ-mediated PD-L1 expression was abolished when compared with its parental counterparts (Fig. 4B). Moreover, when exogenous Id1 (Id1-flag) was expressed in *KRAS*-mutant mouse and human *KRAS*-mutant LUAD cells (Fig. 3C and Supplementary Fig. S6), PD-L1 levels in trametinib-treated *KRAS*-mutant LUAD cells were significantly reduced upon IFN-γ stimulation (Fig. 4B). Representative histograms of the flow cytometry assays are shown in Supplementary Fig. S7. Taken together these results suggest that trametinib stimulates IFN-γ-mediated PD-L1 expression via the downregulation of Id1 in *KRAS*-mutant LUAD cells, which does not occur in TR *KRAS*-mutant LUAD cells.

**Fig. 5:**
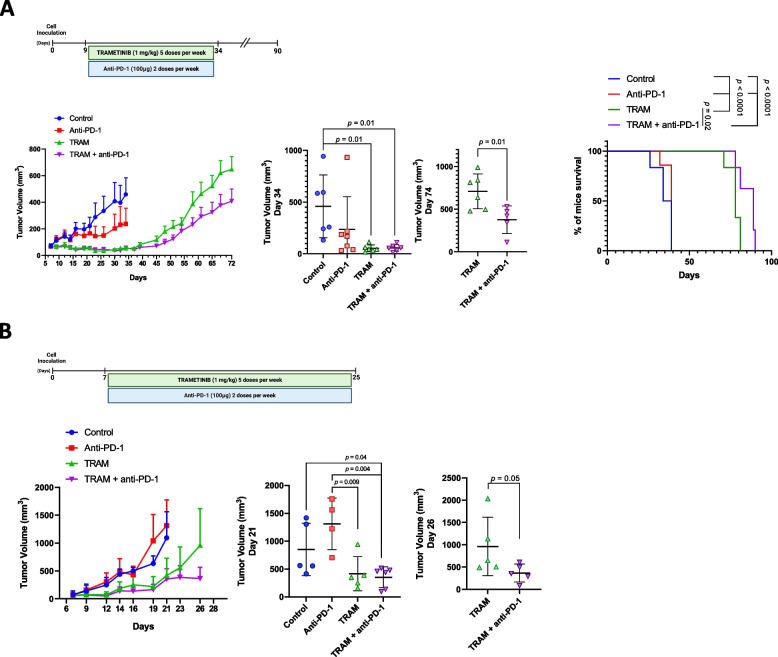
Trametinib combined with PD-1/PD-L1 blockade reduces tumor growth in murine *KRAS*-mutant lung cancer syngeneic models. **A** Upper panel: Schematic of the experiment. Subcutaneously inoculated *KRAS*-mutant 393P cells were allowed to growth in syngeneic mice for 9 days. Tumor-bearing mice were randomized in four groups and treated with anti-PD-1 (Anti-PD-1; twice weekly; *n* = 8 mice per group), trametinib (TRAM; 5 days per week; *n* = 8 mice per group), their combination (TRAM + anti-PD-1; *n* = 8 mice per group) or vehicle (control; *n* = 8 mice per group). Lower panel from left to right: Follow-up of tumor volume over time. Tumor volumes at day 34 of all the experimental groups. Tumor volumes at day 74 of 393P-tumors treated with trametinib alone (TRAM) or in combination with anti-PD-1 (TRAM + anti-PD-1). Kaplan–Meier survival curves for all the experimental groups. **B** Upper panel: Schematic of the experiment. Subcutaneously inoculated *KRAS*-mutant LLC cells were allowed to growth in syngeneic mice for 7 days. Tumor-bearing mice were randomized in four groups and treated with anti-PD-1 (Anti-PD-1; twice weekly; *n* = 6 mice per group), trametinib (TRAM; 5 days per week; *n* = 6 mice per group), their combination (TRAM + anti-PD-1; *n* = 6 mice per group) or vehicle (control; *n* = 6 mice per group). Lower panel from left to right: Follow-up of tumor volume over time. Tumor volumes at day 21 of all the experimental groups. Tumor volumes at day 26 of LLC-tumors treated with trametinib alone (TRAM) or in combination with anti-PD-1 (TRAM + anti-PD-1). Data are expressed as mean ± SD. Comparisons between two experimental groups were performed by two-side *t*-test; for more than two experimental groups one-way ANOVA followed by the Tukey post hoc test was used

In view of the impact of trametinib on PD-L1 expression in tumor cells, we explored the effects of trametinib and anti-PD-1 blockade in two in vivo* KRAS*-mutant LUAD mouse models. The combination of trametinib plus PD-1 blockade significantly reduced 393P tumor growth and improved mice median overall survival when compared with the effect of each treatment alone [Tumor Volume (mm^3^): Day 34: Control: 458.9 ± 303.9, Anti-PD-1: 236.5 ± 315.2, TRAM: 52.9 ± 33.07, TRAM + anti-PD-1: 57.34 ± 33.36; Day 74: TRAM: 709.4 ± 205.2, TRAM + anti-PD-1: 375.8 ± 162.5; Survival (Days): Control: 36.5, Anti-PD-1 39, TRAM: 78, TRAM + anti-PD-1: 89] (Fig. 5A). Similar results for tumor growth were observed for LLC subcutaneous tumors [Tumor Volume (mm^3^): Day 21: Control: 852.4 ± 470.6, Anti-PD-1: 1313 ± 461.1 TRAM: 419.7 ± 306.6, TRAM + Anti-PD-1: 354.7 ± 186.4; Day 26: TRAM: 961 ± 654.7, TRAM + anti-PD-1: 364 ± 201] (Fig. 5B).

**Fig. 6:**
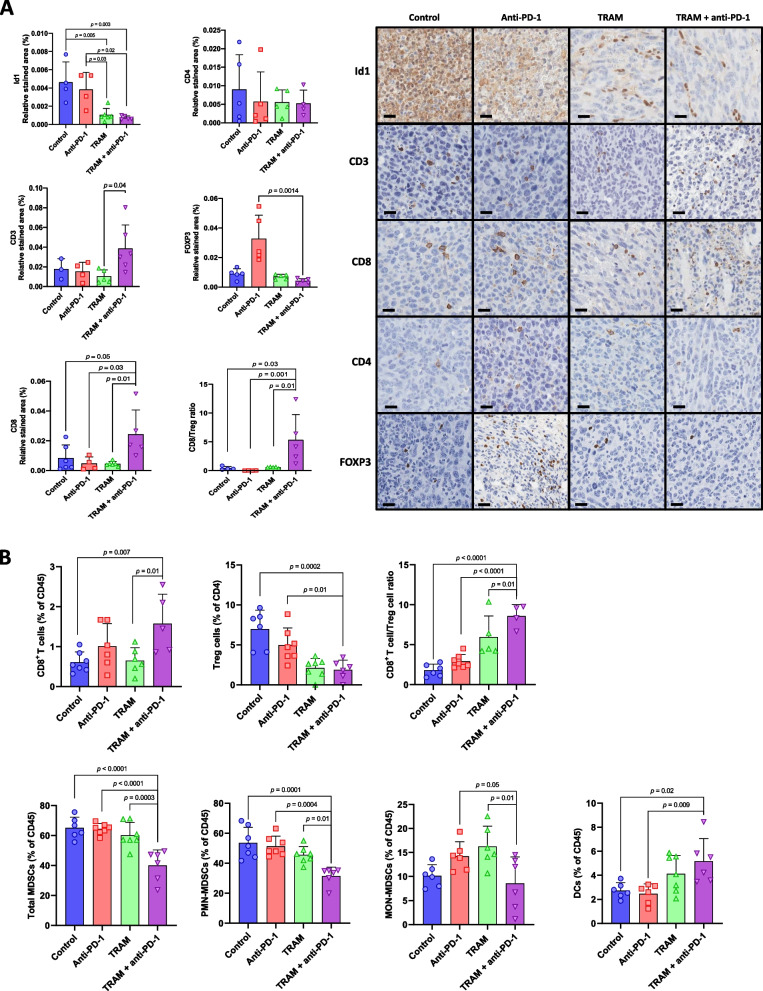
Trametinib in combination with PD-1 blockade increases the frequency of tumor-infiltrating effector T cells and reduces the proportion of immunosuppressive Treg cells and MDSCs within the tumor microenvironment. **A** Left panel: Immunohistochemical quantification of Id1 and tumor-infiltrating CD8^+^ T cells (CD8), CD4^+^ T cells (CD4), Treg cells (Foxp3) in LLC tumors harvested from mice shown in Fig. 5B. CD8 T cells/Treg ratio is also shown. Right panel: Representative images of the immunostainings. Scale bar: 200 μm. **B** Flow cytometric quantification of tumor-infiltrating CD8^+^ T cells (CD45^+^, CD3^+^, CD8^+^, CD44^+^), Treg cells (CD45^+^, CD3^+^, CD4^+^, CD25^+^, Foxp3^+^); total MDSCs (CD45^+^, CD11b^+^, Ly6C^+^), PMN-MDSCs (CD45^+^, CD11b^+^, Ly6C^+^, Ly6G^High^), MON-MDSCs (CD45^+^, CD11b^+^, Ly6C^+^, Ly6G^Low^) and DCs (CD45^+^, CD11c^+^, MHC-II^+^) from LLC tumors harvested from mice shown in Supplementary Fig. S8A. CD8 T cells/Treg ratio is also shown. Data are expressed as the percentage of total leukocytes (CD45), except for Treg cells, which are expressed as the percentage of total CD4^+^ T cells. Data are expressed as mean ± SD. Comparisons between experimental groups were performed by one-way ANOVA followed by the Tukey post hoc test

Next, we characterized by IHC the changes in LLC tumor-infiltrating immune cell populations resulted from trametinib and anti-PD-1 combined treatment. Trametinib and anti-PD-1 combined treatment led to a significant increase in tumor-infiltrating CD3^+^ T cells and CD8^+^ T cells whereas no differences in the frequencies of tumor-infiltrating CD4^+^ T cells were observed. Moreover, the frequency of tumor-infiltrating Foxp3^+^ cells (Treg cells) were significantly reduced in mice treated with trametinib and anti-PD-1 combination. Interestingly, the CD8^+^/Treg cell ratio was significantly increased in the combined treatment group. Of note, as shown in Fig. 1F Id1 expression in tumor tissues was significantly reduced in trametinib-treated mice (Fig. 6A).

We further characterized by flow cytometry the changes in the immune populations resulting from the combined treatment. The experiment was performed 14 days after LLC tumor inoculation (Supplementary Fig. S8A). As observed before, the combined treatment led to an increase of tumor-infiltrating CD8^+^ T cells, a reduction in the proportion of Treg cells and a significant increase of the CD8^+^/Treg cell ratio (Fig. 6B; Supplementary Fig. S8B). Besides, an increase in B cells was observed in the combined treatment (Supplementary Fig S8B) No remarkable differences were observed in the proportions of tumor-infiltrating CD4^+^ T cells and natural killer (NK) cells. No differences in the expression of the T cell exhaustion marker LAG-3 were found in CD4^+^ and CD8^+^ T cells. The exhaustion marker PD-1 was only inhibited in tumor-infiltrating CD4^+^ and CD8^+^ T harvested from anti-PD-1-treated tumors (Supplementary Fig. S8B). Tumor-infiltrating myeloid subpopulations were also analyzed. The combined treatment significantly reduced the proportion of total and polymorphonuclear (PMN) myeloid-derived suppressor cells (MDSCs). No remarkable differences were observed for the proportions of monocytic MDSCs (MON-MDSCs) (Fig. 6B). A significant increase of tumor-infiltrating DCs and M1 macrophages was observed in the combined group (Fig. 6B and Supplementary Fig. S8B). No remarkable differences were observed in the proportions of tumor-infiltrating total and M2 macrophages (Supplementary Fig. S8B).

Taken together, these findings indicate that trametinib reduces Id1 levels to enhance IFN-γ-mediated PD-L1 expression and sensitizes *KRAS*-mutant LUAD tumors to anti-PD-1 treatment. This effect was associated with a less immunosuppressive tumor microenvironment.

### Id1 expression inhibits the antitumor activity of the trametinib and anti-PD-1 combined treatment in LLC tumors

**Fig. 7:**
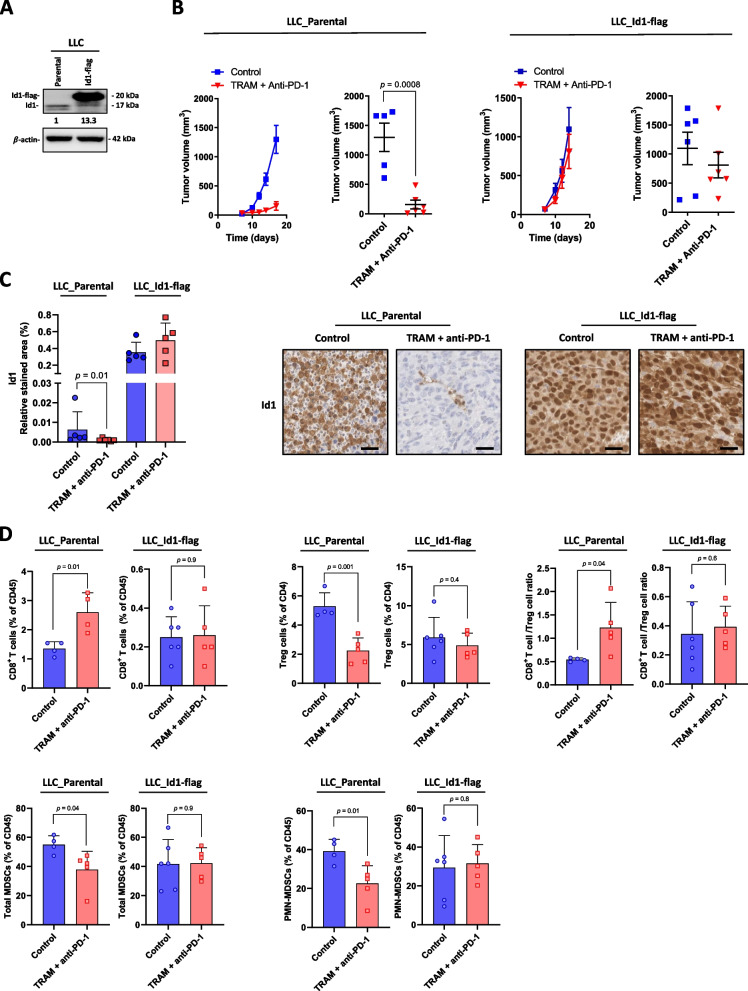
The antitumor activity of the trametinib and anti-PD-1 combined treatment depends on trametinib-mediated Id1 inhibition. **A** Western blot analysis of Id1 protein in parental and LLC Id1-flag LLC cells. β-Actin was used as control. **B** Left panel: Subcutaneous growth of LLC tumors in mice treated with trametinib and anti-PD-1 combination (TRAM + anti-PD-1; TRAM; 5 days per week; anti-PD-1; twice weekly) (*n* = 6 mice per group) or vehicle (control) (*n* = 5 mice per group). The follow-up of tumor size and the tumor volumes at day 14 are shown. Right panel: Subcutaneous growth of LLC Id1-flag tumors in mice treated with trametinib and anti-PD-1 combination (TRAM + anti-PD-1; TRAM; 5 days per week; anti-PD-1; twice weekly) (*n* = 6 mice per group) or vehicle (control) (*n* = 6 mice per group). The follow-up of tumor size and the tumor volumes at day 17 are shown. **C** Immunohistochemical quantification and representative images of Id1 immunostaining in the tumors from the experiment shown in B. Scale bar: 200 μm. **D** Flow cytometric quantification of total tumor-infiltrating CD8^+^ T cells (CD45^+^, CD3^+^, CD8^+^, CD44^+^), Treg cells (CD45^+^, CD3^+^, CD4^+^, CD25^+^, Foxp3^+^), total MDSCs (CD45^+^, CD11b^+^, Ly6C^+^), PMN-MDSCs (CD45^+^, CD11b^+^, Ly6C^+^, Ly6G^High^). CD8 T cells/Treg ratio is also shown. Data are expressed as the percentage of total leukocytes (CD45^+^), except for Treg cells, which are expressed as the percentage of total CD4^+^ T cells. Data are expressed as means ± SD. Comparisons between experimental groups were performed by two-sided *t*-test (parametric) or two-sided Mann–Whitney *U*-test (non-parametric)

We evaluated whether the antitumor effect of the trametinib and anti-PD-1 combined treatment depends on trametinib-mediated Id1 inhibition. For this purpose, we tested the effect of the combined treatment on LLC tumors in which exogenous Id1 (Id1-flag) was expressed (Fig. 7A). As shown in Fig. 7B the combined treatment significantly reduced parental LLC tumor growth. However, this inhibition was abolished in LLC Id1-flag tumors (LLC_Parental (mm^3^): Control: 1300 ± 240.5, TRAM + anti-PD-1: 158 ± 73.7; LLC_Id1-flag: Control: 1097 ± 279, TRAM + anti-PD-1:812.2 ± 220) (Fig. 7B). Of note, LLC Id1-flag tumors grew markedly faster that their parental counterparts. Similarly, to in vitro observations (Fig. 3C), trametinib treatment did not affect Id1 expression levels in LLC Id1-flag tumors whereas a significant Id1 level reduction was observed in parental LLC tumors after such treatment (LLC_Parental: Control: 0.0063 ± 0.0009, TRAM + anti-PD-1: 0.001 ± 0.00007; LLC_Id1-flag: Control: 0.35 ± 0.12, TRAM + anti-PD-1: 0.49 ± 0.2) (Fig. 7C). We also analyzed the proportion of the tumor-infiltrating immune populations in these tumors. Differences in CD8^+^ T cells, total MDSCs, PMN-MDSCs, Treg cells and CD8/Treg cell ratio between control and combined treatment experimental groups were consistent with those shown in Fig. 6B. However, these differences were lost in LLC Id1-flag tumors (Fig. 7D). No remarkable differences were observed in the proportion of tumor-infiltrating CD4^+^ T cells, NK cells, B cells, DCs and tumor-infiltrating macrophages (Supplementary Fig S9).

Overall, the presence of Id1 in LLC tumor cells impairs the antitumor activity of the combined treatment and restores an immunosuppressive tumor microenvironment.

## Discussion

Resistance to the MEK inhibitor trametinib represents a major challenge in the therapeutic management of patients with mutant *KRAS* LUAD. In this study, we found that trametinib treatment downregulates Id1, a transcriptional regulator involved in the oncogenic mutant *KRAS* pathway [24], via proteasome-ubiquitin pathway to sensitize *KRAS*-mutant LUAD tumors to PD-1 blockade immunotherapy. Moreover, our data suggest that Id1 may be involved in the acquired resistance of *KRAS*-mutant LUAD to trametinib*.* These data open up new avenues to treating patients with *KRAS*-mutant lung cancer.

The protumoral phenotype conferred by Id1 expression in *KRAS*-mutant LUAD has been related to the induction of tumor angiogenesis, tumor-associated immunosuppression and metastasis [19, 23, 24]. Consequently, high levels of Id1 are associated with a reduced overall survival and poor prognosis in these patients [22]. This has prompted a series of attempts to pharmacologically target Id1 activity, including the development of antisense molecules [46], peptide aptamers [47] and the development of small-molecules such as AGX51, an Id family antagonist [48, 49]. However, the lack of a catalytic domain and the nuclear localization of Id proteins, make them a difficult pharmacological target and hampered the approval of these inhibitors. Currently, none of those therapeutic strategies against Id1 has been clinically tested in patients. Our results reveal that the use of trametinib may represent an interesting alternative to directly target Id1.

The constitutive activation of the RAS–ERK pathway as a result of *KRAS* mutations has led to the development of MEK inhibitors (MEKi) as a potential therapeutic strategy for the treatment of *KRAS*-mutant cancers [35]. In the present work, we found that trametinib mediated MEK1/2 inhibition downregulates Id1 protein levels in human and murine lung cancer cells, regardless *KRAS*-mutational status. Interestingly, trametinib inhibited *KRAS*-mutant, but not *KRAS*-wild type LUAD cell proliferation. This was also observed after *Id1* genetic silencing [24] supporting the idea that the antitumor effect exerted by trametinib on *KRAS*-mutant LUAD may be mediated by the downregulation of Id1.

The upregulation of *Id1* mRNA expression levels observed after trametinib treatment suggests that trametinib-mediated Id1 inhibition is associated with a post-translational regulatory process. Although autophagy has been proposed as a mechanism of Id1 degradation in neuroblastoma cells [38], our data indicate that the autophagy inhibitor CQ does not affect the trametinib-mediated Id1 downregulation in *KRAS*-mutant LUAD cells. Members of the Id family, like other cell-cycle regulators, are potential substrates of the ubiquitin (Ub)/26 S proteasome system [50, 51]. Our RNA-seq analysis reveals a significant enrichment of pathways related with proteasome-ubiquitin system in trametinib-treated *KRAS*-mutant LUAD cells. Importantly, the blockade of the proteasome abrogated Id1 downregulation in trametinib-treated *KRAS*-mutant LUAD cells. Similarly, in colorectal *KRAS*-mutant cancer cells, trametinib downregulates Mcl-1 levels in a ubiquitin–proteasome-dependent manner through E3 ligase activation [43]. E3 ligases can regulate Id1 degradation through ubiquitination of Id-family members at internal lysine residues and in N-terminal residues [39, 21]. Interestingly, we found that trametinib-mediated Id1 degradation in *KRAS*-mutant LUAD cells was associated with the upregulation of the E3 ubiquitin ligases SMURF-2 and FBXW7. Indeed, the inhibition of SMURF2 with a specific shRNA, partially blocked trametinib-mediated Id1 inhibition. Collectively, these data may suggest that trametinib fuels the proteasome activity to degrade Id1 in *KRAS*-mutant LUAD cells via upregulation of E3 ubiquitin ligases.

There is compelling evidence that some targeted therapies may sensitize tumors to immune-mediated killing, improving ICIs efficacy in NSCLC [17, 52]. Using preclinical experimental models, we have shown that trametinib and PD-1 blockade combined treatment significantly reduced *KRAS*-mutant LUAD tumor growth in a synergistic manner. In this regard, the combined treatment increased the proportions of tumor-infiltrating CD8^+^ T cells and DCs. Moreover, the combined treatment reduced the proportions of the immunosuppressive intratumoral Treg cells, total MDSCs and PMN-MDSCs, whereas the ratio of CD8/Treg was increased. These data indicate that the combined treatment reverses the LLC immunosuppressive microenvironment and promotes a specific antitumor immune response. In concordance with our results, other clinical and preclinical data have demonstrated that MEK1/2 inhibition enhances CD8^+^ T cells activity and reduces the infiltration of MDSC to sensitize tumors to ICIs [12, 53].

Our data indicate that trametinib enhances IFN-γ-mediated PD-L1 expression on *KRAS*-mutant LUAD cell surface via Id1 downregulation. Indeed, trametinib/IFN-γ-mediated PD-L1 expression was abolished when the Id1 levels were restored. Previously, we found that *Id1* downregulation enhances IFN-γ-mediated PD-L1 expression on *KRAS*-mutant LUAD cells [19]. High PD-L1 levels in neoplastic tissues have been associated with enhanced objective response rates to PD-1/PD-L1 blockade in NSCLC tumors [54, 55]. According to our data, an inverse correlation between *Id1* and *PD-L1* mRNA levels was found in *KRAS*-mutant LUAD human tumors [19]. The synergy of MEKi with anti-PD-1/PD-L1 antibodies has been previously reported in NSCLC [12, 56], but none of these studies provide mechanistic evidence for trametinib-mediated sensitization of *KRAS*-mutant LUAD human tumors to PD-1/PD-L1 blockade. In this study, we have identified for the first time that this sensitization depends of the trametinib-mediated Id1 inhibition. In this regard, in LLC cells in which exogenous Id1 (Id1-flag) was expressed independent of trametinib treatment, the trametinib-induced PD-L1 expression upon IFN-γ incubation was abolished and importantly, the trametinib and anti-PD-1 combined treatment did not modify the immunosuppressive tumor microenvironment. Collectively, these data indicate that trametinib sensitizes *KRAS*-mutant LUAD tumors to PD-1/PD-L1 blockade via Id1 inhibition. Trametinib has shown efficacy in multiple types of cancer, such as in *BRAF*-mutant melanoma and NSCLC patients achieving long-term benefit [10, 35]. However, *KRAS*-mutant LUADs eventually develop resistance to treatment [9, 10]. Multiple mechanisms of acquired resistance to MEK inhibition have been reported in different tumor types [57, 58]. One of the best characterized mechanisms involves the reactivation of the MAPK pathway [59, 60]. Our study demonstrates that, in contrast to trametinib sensitive *KRAS*-mutant LUAD, in TR *KRAS*-mutant LUAD cells, trametinib does not downregulate Id1 levels. Importantly, genetic abrogation of *Id1* restored the sensitivity of TR-cells to trametinib suggesting a role for Id1 in the acquired resistance to trametinib in *KRAS*-mutant LUAD. Functional studies in which *KRAS*-mutant LUAD are genetically modified to restore Id1 levels support an active role of Id1 in the resistance to trametinib. Moreover, we demonstrate that in TR *KRAS*-mutant LUAD cells c-Myc and FOSL1 levels, which are well-described mechanisms of targeted therapies against the KRAS pathway [24, 43, 44], are dependent on Id1 expression levels. These results suggest that in *KRAS*-mutant LUAD tumors Id1 may sustain the oncogenic *KRAS*-MAPK signaling pathway through the activation of different kinases or transcription factors. Nevertheless, further investigation is warranted to better understand the role of Id1 in the primary resistance to MEKi.

The possibility of pharmacologically targeting Id1 with trametinib in human cancers could offer a therapeutic alternative for those *KRAS*-mutant LUAD patients who poorly respond to ICIs or develop early acquired resistance. In this regard, an ongoing phase Ib/II clinical trial (NCT03225664) is currently evaluating the safety, efficacy and maximum tolerated dose of trametinib in combination with pembrolizumab in pretreated NSCLC patients. Moreover, the implication of *Id1* gene in trametinib acquired resistance could also serve to design novel immunotherapy combinations, particularly in the context of *KRAS*-mutant tumors.

## Conclusions

In conclusion, we found that trametinib activates the proteasome to reduce Id1 levels in *KRAS*-mutant LUAD tumor cells. The antitumor effects of trametinib in *KRAS*-mutant LUAD cells depend on trametinib-mediated Id1 downregulation. Moreover, we found that Id1 plays a major role in the acquired resistance to trametinib treatment in *KRAS*-mutant LUAD tumor cells. Using *KRAS*-mutant LUAD tumor mouse models we have identified that trametinib-mediated Id1 downregulation sensitizes *KRAS*-mutant LUAD tumors to anti-PD-1/PD-L1 blockade. Our data suggest a prominent role for Id1 in the antitumor activity of trametinib.

### Supplementary Information

**Supplementary Material 1. **

**Supplementary Material 2. **

## Data Availability

No datasets were generated or analysed during the current study.

## Abbreviations

DMSO: Dimethyl sulfoxide
DC: Dendritic cells
H&E: Hematoxylin–eosin
ICIs: Immune checkpoint inhibitors
Id1: Inhibitor of differentiation
IFN-γ: Interferon gamma
IHC: Immunohistochemistry
LUAD: Lung adenocarcinoma
MAPK: Mitogen-activated protein kinases
MDSC: Myeloid-derived suppressor cells
MEKi: MEK inhibitor
MON-MDSC: Monocytic MDSC
NK: Natural killer
NSCLC: Non-small cell lung cancer
PD-1: Programed-cell death protein 1
PD-L1: Programed-cell death-ligand 1
PMN-MDSC: Polymorphonuclear MDSC
TAM: Tumor associated macrophages
TR: Trametinib resistant
TRAM: Trametinib
