# Supplementary material for: Trametinib sensitizes KRAS-mutant lung adenocarcinoma tumors to PD-1/PD-L1 axis blockade via Id1 downregulation
Source: Mol Cancer. 2024 Apr 20;23:78. doi: 10.1186/s12943-024-01991-3 (PMC11031964; doi:10.1186/s12943-024-01991-3)
Supplement: Supplementary file 1 — Supplementary Material 1. [file 12943_2024_1991_MOESM1_ESM.docx]

**Supplementary Fig. S1**

**Supplementary Fig. S1. Flow cytometry gating strategy for the analysis of tumor-infiltrating myeloid cells.** FSC-H/FSC-A representation was used to exclude doublets. Debris and dead cells were excluded by PromoFluor-840 NIR Maleimide staining. Total leukocytes (CD45^+^ cells) were selected and myeloid cells were classified into total MDSCs (CD45^+^, CD11b^+^, Ly6C^+^), PMN-MDSCs (CD45^+^, CD11b^+^, Ly6C^+^, Ly6G^+^), MON-MDSCs (CD45^+^, CD11b^+^, Ly6C^+^, Ly6G^Low^), Tumor associated macrophages (CD45^+^, CD11b^+^, F4/80^+^, MHC-CII^+^), M1 macrophages (CD45^+^, CD11b^+^, F4/80^+^, MHC-CII^+^, CD38^+^), M2 macrophages (CD45^+^, CD11b^+^, F4/80^+^, MHC-CII^+^, CD206^+^) and DCs (CD45^+^, CD11c^+^, MHC-CII^+^).

**Supplementary Fig. S2**

**Supplementary Fig. S2. Flow cytometry gating strategy for the analysis of tumor-infiltrating lymphocytes.** FSC-H/FSC-A representation was used to exclude doublets. Debris and dead cells were excluded by PromoFluor-840 NIR Maleimide staining. Total leukocytes (CD45^+^cells) were selected and lymphocytes were selected by FSC-A/SSC-A representation. Lymphocytes were classified into B cells (CD45^+^, CD19^+^), NK cells (CD45^+^, CD3^-^, NK1.1^+^), CD8^+^ T cells (CD45^+^, CD3^+^, CD8^+^, CD44^+^), CD4^+^ T cells (CD45^+,^ CD3^+^, CD4^+^) and Treg cells (CD3^+^, CD4^+^, CD25^+^, Foxp3^+^).

**Supplementary Fig. S3**

**Supplementary Fig. S3. Effect of trametinib in CMT167 and LLC tumor growth.** Tumor-bearing mice (n=8 mice per group) were treated with trametinib (TRAM, 5 doses per week) or vehicle. A) Left: CMT167 tumor growth. Right: CMT167 tumor volume at day 19. B) Left: LLC tumor growth. Right: LLC tumor volume at day 19. Data are expressed as mean ± SD. Comparisons between experiential groups were performed by two-sided *t*-test.

**Supplementary Fig. S4**

**Supplementary Fig. S4. Autophagy inhibition does not affect trametinib-mediated Id1 inhibition.** A) Western blot analysis of P62, phosphorylated (phospho-) and total p42/44 MAPK, Id1, LC3-I and LC3-II in *KRAS*-mutant mouse (upper panel) and human (lower panel) LUAD cell lines treated with trametinib (TRAM) (100nM in murine cells and 500nM in human cells) for 72 hours or vehicle (Control), in the presence or not of the autophagy inhibitor hydroxychloroquine (CQ) (30 μM) for 6h and 16h. GAPDH and HSP90 were used as controls for mouse and human cell lines, respectively. B) Expression of the E3 ubiquitin ligases *SMURF2* and *FBXW7* in *KRAS*-mutant murine (CMT167 and KLA) LUAD cells treated with trametinib (TRAM) 100 nM for 72 or vehicle (Control). These data were obtained from the RNA-seq analysis. In western blot analyses, relative optical density is indicated underneath each lane. Data are expressed as means ± SD. Comparisons between experiential groups were performed by two-sided *t*-test.

**Supplementary Fig. S5**

**Supplementary Fig. S5.** A) Cell proliferation curves of parental and KRAS-mutant mouse (left panel) and human (right panel) LUAD cell lines treated with trametinib. Trametinib IC50 are indicated in the figure. B) Expression of the E3 ubiquitin ligase *SMURF2* (left) and *Id1* (right) in parental CMT167 treated with trametinib (TRAM) 100 nM and TR CMT167 cells. These data were obtained from the RNA-seq analysis. C) Western blot analysis of Id1 in TR *KRAS*-mutant mouse (left) and human (right) cells. Id1 was silenced in TR cells using lentiviral transduced shRNA (sh1-Id1 and sh2-Id1 in mice and sh-Id1 in human) or with a scrambled sequence (Control). β-Actin was used as control. D) Upper panel: Western blot analysis of Id1 (17 kDa) and Id1-flag (20 kDa) proteins in 393P transfected cells. Control cells were transfected with a GFP cDNA expressing vector (control). E) treated with trametinib (TRAM) 100nM for 72 hours or vehicle (Control). β-actin was used as control. Lower panel: Effects of exogenous Id1 (Id1-flag)-transduction in the survival of *KRAS*-mutant mouse 393P trametinib-treated tumor cells. Trametinib IC50 is indicated in the figure. In western blot analyses, relative optical density is indicated underneath each lane. Data are expressed as means ± SD. Comparisons between experiential groups were performed by two-sided *t*-test.

**Supplementary Fig. S6**

**Supplementary Fig. S6. Effect of trametinib in Id1-flag KRAS-mutant cells in Id1 levels.** Western blot analysis of Id1 (17 kDa) and Id1-flag (20 kDa) proteins in CMT167, A549 and H2030 transfected cells. in GFP cDNA expressing vector (control) and in exogenous Id1 (Id1-flag)-transduced *KRAS*-mutant mouse (left) and human (right) Cells were treated with trametinib (TRAM) (100nM in murine cells and 500nM in human cells) for 72 hours or vehicle (Control). β-actin and GAPDH were used as control in mouse and human cells, respectively. Relative optical density is indicated underneath each lane.

**Supplementary Fig. S7**

**Supplementary Fig. S7. Trametinib upregulates PD-L1 expression through Id1 downregulation in *KRAS*-mutant LUAD cells.** Flow cytometry histograms from the experiment shown in Fig. 4

**Supplementary Fig. S8**

**Supplementary Fig. S8. Effects of the combined treatment in the LLC tumor infiltrate.** A) Left panel: Tumor growth of LLC inoculated subcutaneously in syngeneic mice treated with anti-PD-1 (Anti-PD-1; twice weekly; n=6), trametinib (TRAM; 5 days per week; n=6), their combination (TRAM + anti-PD-1; n=6) or vehicle (control; n=6) (left). Right panel: Tumor volumes at day 14 of all the experimental groups. B) Upper panel: Flow cytometric quantification of tumor-infiltrating CD4^+^ T cells (CD45^+,^ CD3^+^, CD4^+^), NK cells (CD45^+^, CD3^-^, NK1.1^+^) and B cells (CD45^+^, CD19^+^). Middle panel: PD-1 and LAG-3 expression represented as median fluorescence intensity (MFI) in CD8^+^ T (Left) and CD4^+^ T cells (right). Lower panel: Flow cytometric quantification of tumor-infiltrating tumor-associated macrophages (TAMs) (CD45^+^, CD11b^+^, F4/80^+^, MHC-CII^+^), M1 macrophages (CD45^+^, CD11b^+^, F4/80^+^, MHC-CII^+^, CD38^+^), M2 macrophages (CD45^+^, CD11b^+^, F4/80^+^, MHC-CII^+^, CD206^+^) from the experiment shown in A. Data are expressed as the percentage of total leukocytes (CD45). Data are expressed as means ± SD. Comparisons between experimental groups were performed by one-way ANOVA test followed by the Tukey *post hoc* test.

**Supplementary Fig. S9**

**Supplementary Fig. S9. Effects of the combined treatment in the LLC Id1-flag tumor infiltrate.** Upper panel: Flow cytometric quantification of tumor-infiltrating CD4^+^ T cells (CD45^+,^ CD3^+^, CD4^+^), B cells (CD45^+^, CD19^+^) and NK cells (CD45^+^, CD3^-^, NK1.1^+^). Middle panel: Flow cytometric quantification of tumor-infiltrating Tumor associated macrophages (TAMs) (CD45^+^, CD11b^+^, F4/80^+^, MHC-CII^+^), M1 macrophages (CD45^+^, CD11b^+^, F4/80^+^, MHC-CII^+^, CD38^+^), M2 macrophages (CD45^+^, CD11b^+^, F4/80^+^, MHC-CII^+^, CD206^+^). Lower panel: low cytometric quantification of DCs (CD45^+^, CD11c^+^, MHC-CII^+^) from the experiment shown in Fig.7. Data are expressed as the percentage of total leukocytes (CD45). Data are expressed as means ± SD. Comparisons between experimental groups were performed by two-sided *t*-test.
