# Supplementary material for: Trametinib sensitizes KRAS-mutant lung adenocarcinoma tumors to PD-1/PD-L1 axis blockade via Id1 downregulation
Source: Mol Cancer. 2024 Apr 20;23:78. doi: 10.1186/s12943-024-01991-3 (PMC11031964; doi:10.1186/s12943-024-01991-3)
Supplement: Supplementary file 2 — Supplementary Material 2. [file 12943_2024_1991_MOESM2_ESM.docx]

**Supplementary Table S1. Antibodies used for western blot analysis.**

| **Antibodies** | **Source** | **Reference** | **Clone** | **Dilution** |
| --- | --- | --- | --- | --- |
| Rabbit anti-Total P62 | Cell Signaling | 39749 | D1Q5S | 1:1000 |
| Rabbit anti-LC3 A/B | Cell Signaling | 12741 | D3U4C | 1:1000 |
| Mouse anti-β-ACTIN | Sigma-Aldrich | A5441 | AC-15 | 1:5000 |
| Mouse anti-β-TUBULIN | Sigma-Aldrich | T4026 | TUB 2.1 | 1:5000 |
| Rabbit anti-Id1 | BioCheck | HPAB-0309-YC | BCH-1/ 195-14 | 1:1000 |
| Rabbit anti-Total ERK 1/2 | Cell Signaling | 4695 | 137F5 | 1:1000 |
| Rabbit anti- pERK 1/2 | Cell Signaling | 4370 | D13.14.4E | 1:1000 |
| Rabbit anti-GAPDH | Cell Signaling | 5174 | D16H11 | 1:5000 |
| Mouse anti-HSP90 | Santacruz | Sc-13119 | F-8 | 1:5000 |
| Rabbit anti-FRA1 (FOSL1) | Cell Signaling | 5281S | D80B4 | 1:1000 |
| Rabbit anti-cMYC | Cell Signaling | 5605S | D84C12 | 1:1000 |
| Rabbit anti-SMURF2 | Cell Signaling | 12024 | D8B8 | 1:1000 |
| Mouse anti-FBXW7 | Santacruz | Sc-293423 | 3D1 | 1:500 |

**Supplementary Table S2. Primer sequences used for real-time PCR analysis.**

| **Gene** | **Sequence** | |
| --- | --- | --- |
| *Id1* | Fw | GGAGTCTGAAGTCGGGACCA |
|  | Rv | CTGGAACACATGCCGCCT |
| *ID1* | Fw | GCGAGATCAGTGCCTTGG |
|  | Rv | CTCCTGAAGGGCTGGAGTC |
| *β-ACTIN* | Fw | CCTCGCCTTTGCCGATCC |
|  | Rv | CGCGGCGATATCATCATCC |
| *β-Actin* | Fw | GCAAGCAGGAGTACGATGAGT |
|  | Rv | AGGGTGTAAAACGCAGCTCAG |
| *Smurf-2* | Fw | CGCCTGACAGTACTCTGTGCAA |
|  | Rv | TGCCCAGAACCATCAACTACC |
| *SMURF-2* | Fw | CATGTCTAACCCCGGAGGC |
|  | Rv | GCAAATGGATCAGGAAGTCGG |
| *Fbxw7* | Fw | CCGCCTCAGCCCGAA |
|  | Rv | AAAGTCTCCTTGATAGCTGAGATT |
| *FBXW7* | Fw | GCCTCTACCACATCAAACTGTG |
|  | Rv | AAAGAGCGGACCTCAGAACC |

**Supplementary Table S3. Antibodies used for flow cytometry analysis.**

| **Antibodies** | **Laser** | **Fluorochrome** | **Clone** | **Source** | **Reference** | **Dilution** |
| --- | --- | --- | --- | --- | --- | --- |
| CD3e | Red Laser (633 nm) | PE**-A** | (20/70) | BioLegend | 553063 | 1:200 |
| αNK1.1 | Red Laser (633 nm) | R660-APC**-A** | (PK136) | BioLegend | 108710 | 1:20 |
| αCD45 | Red Laser (633 nm) | R763-APCA750-A | (30-F11) | BioLegend | 103116 | 1:500 |
| αLy6G | Red Laser (633 nm) | R712-APCA700-A | (1A8) | BioLegend | 127622 | 1:200 |
| αCD62L | Red Laser (633 nm) | R712-APCA700-A | (MEL-14) | BioLegend | 104426 | 1:800 |
| αCD38 | Blue Laser (488 nm) | B690-PCP5,5-A | 90 | BioLegend | 102722 | 1:160 |
| αCD25 | Blue Laser (488 nm) | B690-PCP5,5-A | (PC61) | BioLegend | 102028 | 1:50 |
| αCD206 | Blue Laser (488 nm) Green Laser (532 nm)/Yellow-Green Laser (561 nm) | Y763-PC7-A | (C068C2) | BioLegend | 141720 | 1:40 |
| αFoxP3 | Blue Laser (496nm) | Y763-PC7-A | (3G3) | Abcam | ab210232 | 1:320 |
| αPD-L1 | Violet Laser (405 nm) | V450-PB-A | (10F.9G2) | BioLegend | 124315 | 1:80 |
| αGzB | Violet Laser (405 nm) | V450-PB-A | (GB11) | BioLegend | 515408 | 1:20 |
| αLy6C | Violet Laser (405 nm) | V525-KrO-A | (HK1.4) | BioLegend | 128033 | 1:200 |
| αCD44 | Violet Laser (405 nm) | V525-KrO-A | (IM7) | BioLegend | 103044 | 1:200 |
| αF4/80 | Violet Laser (405 nm) | V610-A | (BM8) | BioLegend | 123133 | 1:80 |
| αPD-1 | Violet Laser (405 nm) | V610-A | (29F.1A12) | BioLegend | 135220 | 1:80 |
| αMHC-C2 | Violet Laser (405 nm) | V660-A | (M5/114.15.2) | BioLegend | 107641 | 1:80 |
| αLAG3 | Violet Laser (405 nm) | V660-A | (C9B7W) | BioLegend | 125227 | 1:400 |
| αCD11c | Ultraviolet Láser (355 nm) | UV405-A | (HL3) | BD Bioscience | 564080 | 1:80 |
| αCD8 | Ultraviolet Láser (355 nm) | UV405-A | (53-6.7) | BD Bioscience | 563786 | 1:200 |
| CD4 | Ultraviolet Láser (355 nm) | UV525-A | (GK1.5) | BD Bioscience | 612952 | 1:400 |
| CD11b | Ultraviolet Láser (355 nm) | UV675**-A** | (M1/70) | BD Bioscience | 565080 | 1:160 |
| CD19 | Ultraviolet Láser (355 nm | UV675**-A** | (1D3) | BD Bioscience | 565076 | 1:200 |
| PD-L1 (Human) | Red Laser (633 nm) | PE**-A** | 10F.29E.2A3 | BioLegend | 329705 | 1:500 |
| PD-L1 (Mouse) | Red Laser (633 nm) | PE**-A** | 10F.9G2 | BioLegend | 124307 | 1:500 |
